# *PTEN* Deletions Are Associated With Tumor Progression But Unrelated to Patient Prognosis in Muscle‐Invasive Urothelial Bladder Carcinomas: A Large Multi‐Center Validation Study on 2710 Urothelial Bladder Carcinomas

**DOI:** 10.1002/gcc.70105

**Published:** 2026-01-24

**Authors:** Martina Kluth, Henning Plage, Kira Furlano, Sebastian Hofbauer, Sarah Weinberger, Annika Fendler, Bernhard Ralla, Simon Schallenberg, Sefer Elezkurtaj, Maximilian Lennartz, Andreas H. Marx, Henrik Samtleben, Margit Fisch, Michael Rink, Marcin Slojewski, Krystian Kaczmarek, Thorsten Ecke, Nico Adamini, Joachim Weischenfeldt, Henrik Zecha, Ronald Simon, Guido Sauter, Thorsten Schlomm, David Horst, Sarah Minner

**Affiliations:** ^1^ Institute of Pathology University Medical Center Hamburg‐Eppendorf Hamburg Germany; ^2^ Department of Urology, Charité – Universitätsmedizin Berlin, Corporate Member of Freie Universität Berlin Humboldt‐Universität Zu Berlin and Berlin Institute of Health Berlin Germany; ^3^ Institute of Pathology, Charité – Universitätsmedizin Berlin, Corporate Member of Freie Universität Berlin Humboldt‐Universität Zu Berlin and Berlin Institute of Health Berlin Germany; ^4^ Department of Pathology Academic Hospital Fuerth Fuerth Germany; ^5^ Department of Urology University Medical Center Hamburg‐Eppendorf Hamburg Germany; ^6^ Department of Urology Marienhospital Hamburg Hamburg Germany; ^7^ Department of Urology and Urological Oncology Pomeranian Medical University Szczecin Poland; ^8^ Department of Urology Helios Hospital Bad Saarow Bad Saarow Germany; ^9^ Department of Urology Albertinen Hospital Hamburg Germany; ^10^ Biotech Research & Innovation Center (BRIC) University of Copenhagen Copenhagen Denmark; ^11^ Finsen Laboratory Rigshospitalet Copenhagen Denmark

**Keywords:** FISH, muscle‐invasive, prognosis, PTEN deletions, tissue microarray, urothelial bladder cancer

## Abstract

The tumor suppressor gene *PTEN* plays an important role in many cancer types. Mechanism of *PTEN* inactivation includes gene mutations and deletions. In this large multi‐center study, we analyzed the impact of *PTEN* deletions on tumor aggressiveness, patient prognosis, and p53 and p16 alterations, especially in muscle‐invasive urothelial bladder carcinomas to expand the results from our previous study on 686 pTa to pT4 urothelial bladder carcinomas. The *PTEN* copy number status was analyzed by fluorescence in situ hybridization (FISH) on more than 2700 urothelial bladder carcinomas in a tissue microarray format. *PTEN* deletion data were compared with clinico‐pathological parameters in pTa and pT2‐4 carcinomas and clinical outcomes in pT2‐4 carcinomas, immunohistochemical p16 and p53 expression, and *TP53* copy number status measured by FISH from previous studies. *PTEN* deletions occurred in 18.8% of 1854 analyzable carcinomas, including 17.6% heterozygous and 1.2% homozygous deleted tumors. The *PTEN* deletion rate increased markedly from pTaG2 low‐grade (3.1%), to pTaG2 high‐grade (4.5%) and pTaG3 (20.7%, *p* < 0.0001) carcinomas, and was 23.8% in pT2‐4 carcinomas (*p* < 0.0001 for pTa vs. pT2‐4). In pT2‐4 cancers, *PTEN* deletions were unrelated to histopathological parameters of tumor aggressiveness and patient outcome. *PTEN* deletions were significantly associated with parameters of p53 alterations and p16 overexpression. It is concluded that *PTEN* deletions accumulate with grade progression in non‐invasive urothelial carcinomas of the urinary bladder. The absence of a prognostic role of *PTEN* deletions in pT2‐4 urothelial carcinomas is in line with our notorious inability to predict the clinical course of these tumors by only one morphological or molecular feature.

**Abbreviations**
CGH: comparative genomic hybridization
FISH: fluorescence in situ hybridization
IHC: immunohistochemistry
L: lymphatic invasion
LOH: loss of heterozygosity
pN: pathological lymph node status
pT: pathological tumor stage
TMA: tissue microarray
V: venous invasion

## 1 Introduction

Urothelial bladder carcinoma is one of the 10 most common malignant tumor types worldwide [1]. In about 80% of patients, the diagnosis of a low‐grade non‐invasive (pTa) or minimally invasive (pT1) carcinoma is made. These urothelial bladder cancers are characterized by a good prognosis and can be removed by transurethral resection. On the other hand, approximately 20% of all urothelial bladder cancers will further progress to muscle‐invasive cancers [2] and almost 50% of these patients will develop early metastasis and eventually die from their disease despite adequate treatment involving neoadjuvant chemotherapy plus radiotherapy or radical cystectomy [3]. Therefore, it is hoped that a better understanding of the molecular features underlying disease progression will eventually lead to better prognosis prediction and better therapies of urothelial bladder carcinomas.

*PTEN* (phosphatase and tensin homolog deleted in chromosome 10) is a dual specific phosphatase with a role in cell cycle regulation, double‐strand break repair, genomic stability, and chromatin remodeling inside the nucleus [4, 5, 6, 7]. It negatively regulates the phosphatidylinositol 3‐kinase (PI3K) signaling pathway [8, 9]. Suppression or inactivation of PTEN leads to elevated phosphatidylinositol (3,4,5)‐trisphosphate (PIP3) protein levels, thus activation of AKT serine/threonine kinase (AKT) as well as downstream cascades such as mammalian target of rapamycin (mTOR) [10], and therefore, to increased cell survival, cell growth, and cell proliferation, and decreased apoptosis [11, 12, 13]. In cancer, PTEN inactivation or reduced expression is mostly due to mono‐ or biallelic *PTEN* deletion and/or gene mutation as well as epigenetic changes (summarized in [14]). PTEN downregulation is associated with adverse tumor features and poor patient prognosis in many cancer types including prostate [15], kidney [16], breast [17], and lung cancer [18]. In urothelial carcinoma of the urinary bladder, PTEN inactivation seems to occur frequently although its prevalence and clinical relevance is not fully clarified. PTEN aberrations have been found in 7%–80% of urothelial carcinoma of the bladder [19, 20] with some studies suggesting associations with advanced stage and grade, while others found no relationship to tumor phenotype [21, 22, 23, 24, 25, 26, 27, 28]. In our own previous *PTEN* deletion study on 686 pTa to pT4 urothelial bladder carcinomas we found a relationship of *PTEN* deletion with unfavorable tumor features and patient prognosis in early pTa and pT1 urothelial bladder carcinomas but not in pT2 tumors [21].

The aim of our study was to expand the results of our previous study to clarify the prevalence and clinical relevance of *PTEN* deletions, especially in muscle‐invasive urothelial bladder carcinomas, and to examine the relationship of *PTEN* deletions with alterations of p16 and p53 in a large multi‐center study. Therefore, we analyzed *PTEN* copy number status by FISH on more than 2700 urothelial carcinomas in a tissue microarray (TMA) format and compared the results with histopathological parameters of disease progression in non‐muscle‐invasive and muscle‐invasive carcinomas and patient outcome as well as p16 and p53/*TP53* status from a previous study in muscle‐invasive urothelial bladder carcinomas.

## 2 Material and Methods

### 2.1 Tissue Microarrays (TMA)

The TMAs used in this study were first employed in a study on the prognostic role of GATA3 expression in bladder cancer [29]. The TMAs contained one sample each from 2710 urothelial bladder tumors archived at the Institute of Pathology, University Hospital Hamburg, Germany, Institute of Pathology, Charité Berlin, Germany, Department of Pathology, Academic Hospital Fuerth, Germany, or Department of Pathology, Helios Hospital Bad Saarow, Germany, and/or treated at Department of Urology, University Hospital Hamburg, Germany, Department of Urology, Charité Berlin, Germany, Department of Urology, Helios Hospital Bad Saarow, Germany, Department of Urology, Albertinen Hospital, Hamburg, Germany, and Department of Urology and Urological Oncology, Pomeranian Medical University, Szczecin, Poland. Patients at each center were treated according to the guidelines at the time. In brief, patients with pTa disease underwent a transurethral bladder tumor resection with or without postoperative or adjuvant instillation therapy. Patients with pT2–pT4 disease were treated by radical cystectomy. Available histopathological data including tumor stage (pT), grade, status of venous (V) and lymphatic (L) invasion, and lymph node status (pN) are shown in Table 1. Clinical follow up data were available from 709 patients with pT2‐4 carcinomas treated by cystectomy as follows: Overall survival: time between cystectomy and death (709 patients, median: 13 months, range: 1–176 months), recurrence‐free survival: time between cystectomy and recurrence (252 patients, median: 11 months, range: 1–75 months), and cancer‐specific death: time between cystectomy and cancer‐specific death (252 patients, median: 15 months, range: 1–77 months). None of these tumors were included in our previous *PTEN* deletion study from Cordes et al. [21]. Immunohistochemistry (IHC) data on p16 and p53 expression and FISH data on *TP53* copy number status were available from previous studies [30, 31]. All tissues were fixed in 4% buffered formalin and then embedded in paraffin. The TMA manufacturing process has previously been described in detail [32, 33]. In brief, one tissue spot (diameter: 0.6 mm) per patient was used.

**TABLE 1:** Patient cohort.

	Study cohort on TMA (*n* = 2710)
Follow up	
Months	709
Mean	24.5
Median	13
Tumor stage	
pTa	887 (39.2%)
pT2	462 (20.4%)
pT3	615 (27.2%)
pT4	298 (13.2%)
Tumor grade	
G2	820 (30.6%)
G3	1858 (69.4%)
Lymphnode metastasis	
pN0	734 (62.0%)
pN+	449 (38.0%)
Resection margin	
R0	595 (80.6%)
R1	143 (19.4%)
Lymphatic invasion	
L0	275 (49.5%)
L1	281 (50.5%)
Venous invasion	
V0	450 (74.4%)
V1	155 (25.6%)

*Note:* Percent in the column “study cohort on TMA” refers to the fraction of samples across each category. Numbers do not always add up to 2710 in the different categories because of cases with missing data.

### 2.2 Fluorescence In Situ Hybridization (FISH)

**FIGURE 1:**
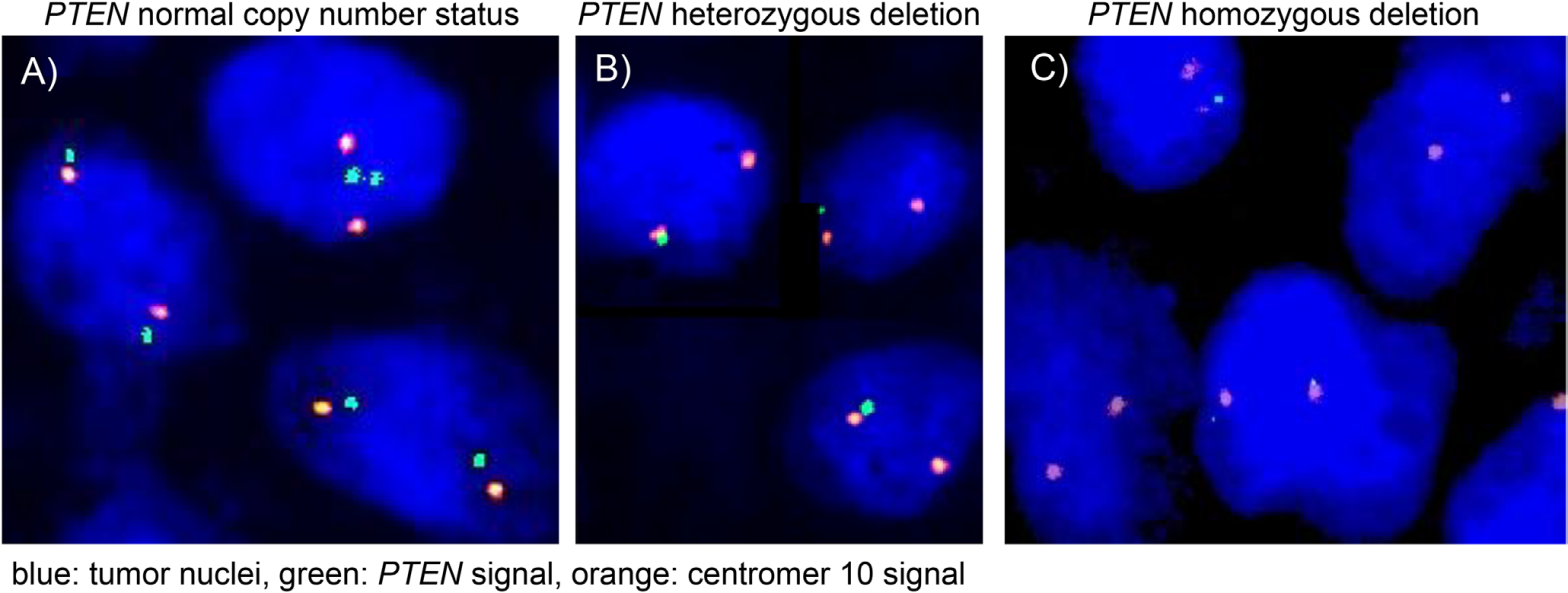
Examples of *PTEN* copy number status measured by fluorescence in situ hybridization. (A) *PTEN* non‐deleted with two green *PTEN* and two orange centromere 10 signals in the tumor cell nuclei, (B) *PTEN* heterozygous deleted with one green *PTEN* and two orange centromere 10 signals in the tumor cell nuclei, and (C) *PTEN* homozygous deleted with absence of green *PTEN* signals in the tumor nuclei and presence of orange centromere 10 signals in the tumor nuclei and green and orange signals in one adjacent normal cell nuclei.

Five micrometer TMA sections were deparaffinized with xylol, rehydrated through a graded alcohol series and exposed to heat‐induced denaturation for 10 min in a water bath at 99°C in P1 pretreatment solution (Agilent Technologies, Santa Clara, CA, USA; #K5799). For proteolytic treatment, slides were added to VP2000 protease buffer (Abbott, North Chicago, IL, USA; #2J.0730) for 200 min at 37°C in a water bath. A commercial FISH probe kit containing both *PTEN* gene specific and corresponding centromere 10 probes was utilized for copy number detection of *PTEN* (ZytoLight SPEC PTEN/CEN 10 Dual Color Probe, Zytovision, Bremerhaven, Germany; # Z‐2078). Hybridization was performed overnight at 37°C in a humidified chamber. Posthybridization washes were done according to the manufacturer's direction (Agilent Technologies, Santa Clara, CA, USA; #K5799). Nuclei were counterstained with 125 ng/mL 4′,6‐diamino‐2‐phenylindole in antifade solution (Biozol; Eching, Germany; #VEC‐H‐1200). Stained tissues were manually interpreted with an epifluorescence microscope and copy numbers of *PTEN* and centromere 10 were estimated for each tissue spot as previously described [21]. Presence of fewer *PTEN* signals than centromere 10 signals in at least 60% of all tumor nuclei in a tumor spot was considered a heterozygous deletion. Absence of *PTEN* signals in the presence of centromere 10 signals in all tumor nuclei and presence of *PTEN* signals in normal cell nuclei was considered a homozygous deletion [15, 21]. All other tumors were considered non‐deleted. Figure 1 gives examples of deleted and non‐deleted tumors. Tissue spots without any detectable *PTEN* signals in all tumor and normal cell nuclei were excluded from the analysis because of a lack of an internal control for successful hybridization.

### 2.3 Statistics

Statistical calculations were performed with JMP18 software (SAS, Cary, NC, USA). Contingency tables and the chi^2^‐test were performed to search for associations between *PTEN* copy number status and *TP53* copy number status, p16 and p53 immunostaining as well as parameters of the tumor phenotype. Survival curves were calculated according to Kaplan–Meier. The Log‐Rank test was applied to detect significant differences between groups.

## 3 Results

### 3.1 Technical Issues

A total of 1854 of 2710 (68.4%) urothelial carcinoma tissue spots were informative in our *PTEN* FISH analysis. Reasons for non‐informative cases (856 spots; 31.6%) included insufficient FISH with absence of unequivocal *PTEN* and/or centromere 10 signals in cancer and non‐neoplastic nuclei, lack of tissue spots or absence of unequivocal cancer nuclei in the TMA spot.

### 3.2 *PTEN* Deletion in Urothelial Carcinomas

**FIGURE 2:**
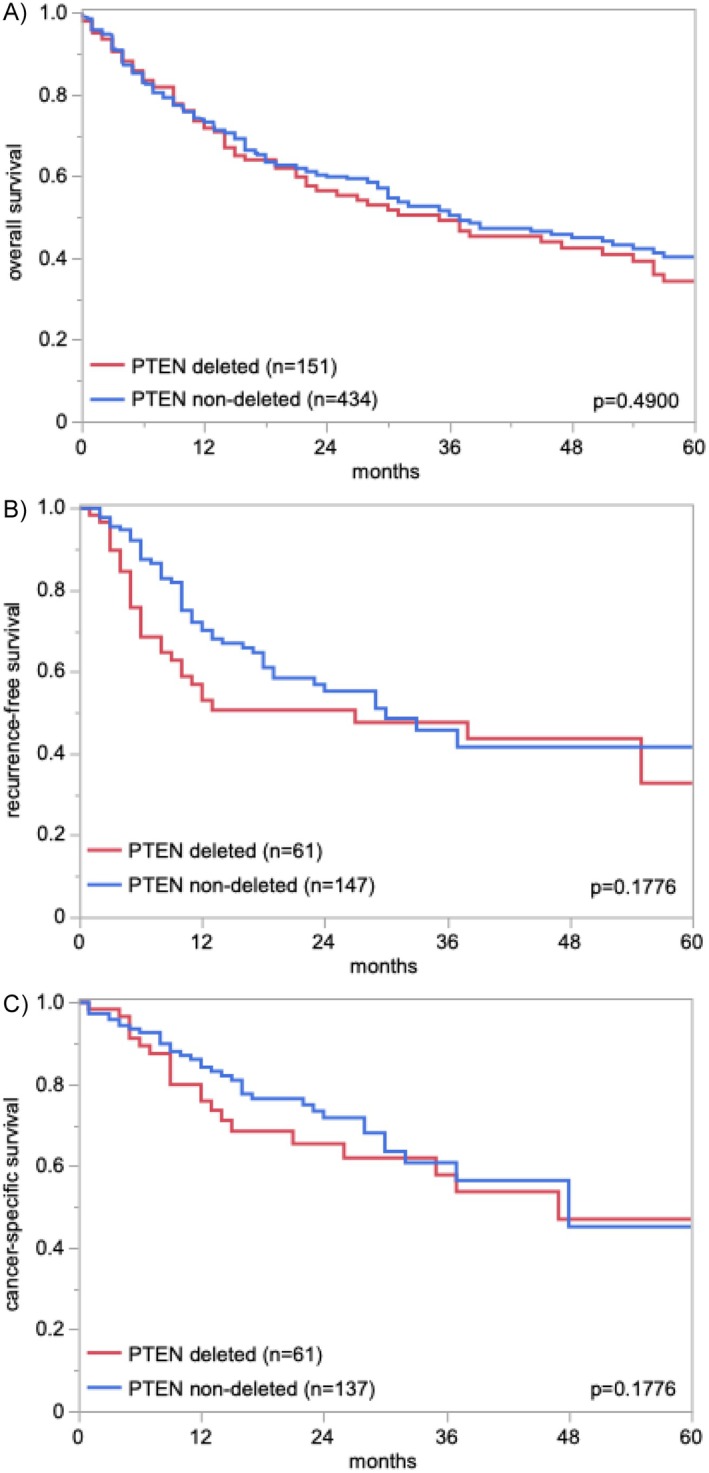
*PTEN* copy number status and patient prognosis.

Deletions of the *PTEN* loci were detectable in 348 (18.8%) of the 1854 analyzable carcinomas including 326 (17.6%) heterozygous and 22 (1.2%) homozygous *PTEN* deleted cancers. The fraction of *PTEN* deleted tumors increased from pTaG2 low‐grade (3.1%), to pTaG2 high‐grade (4.5%) and pTaG3 (20.7%, *p* < 0.0001) carcinomas, and was 23.8% in pT2‐4 carcinomas (*p* < 0.0001 for pTa vs. pT2‐4; Table 2). In pT2‐4 cancers, *PTEN* deletions were unrelated to established parameters of tumor aggressiveness including pT, pN, lymphatic and venous invasion (Table 2) as well as to patient overall survival (*p* > 0.5; Figure 2). Comparable results were found if heterozygous and homozygous deletion were separately analyzed (Table S1).

**TABLE 2:** *PTEN* copy number status and tumor phenotype.

	*n*	PTEN (10q23) copy number status		*p*
		Non‐deleted	Deleted	
All cancers	1854	81.2	18.8	
pTa G2 low	317	96.9	3.2	< 0.0001
pTa G2 high	133	95.5	4.5	
pTa G3	53	79.3	20.8	
pT2	359	76.3	23.7	0.8417^a^
pT3	457	76.2	23.9	
pT4	226	74.3	25.7	
G2	88	80.7	19.3	0.3107^a^
G3	1239	76.0	24.0	
pN0	538	75.3	24.7	0.9444^a^
pN+	349	75.1	24.9	
R0	440	75.7	24.3	0.4862^a^
R1	105	72.4	27.6	
L0	193	75.1	24.9	0.9581^a^
L1	211	75.4	24.6	
V0	326	75.8	24.2	0.7920^a^
V1	113	77.0	23.0	

Abbreviations: G, grade; L, lymphatic invasion; pN, pathological lymph node status; pT, pathological tumor stage; R, resection margin status; V, venous invasion.

^a^ Only in pT2–4 urothelial carcinoma.

### 3.3 *PTEN* Deletion and p53/p16 Status

**FIGURE 3:**
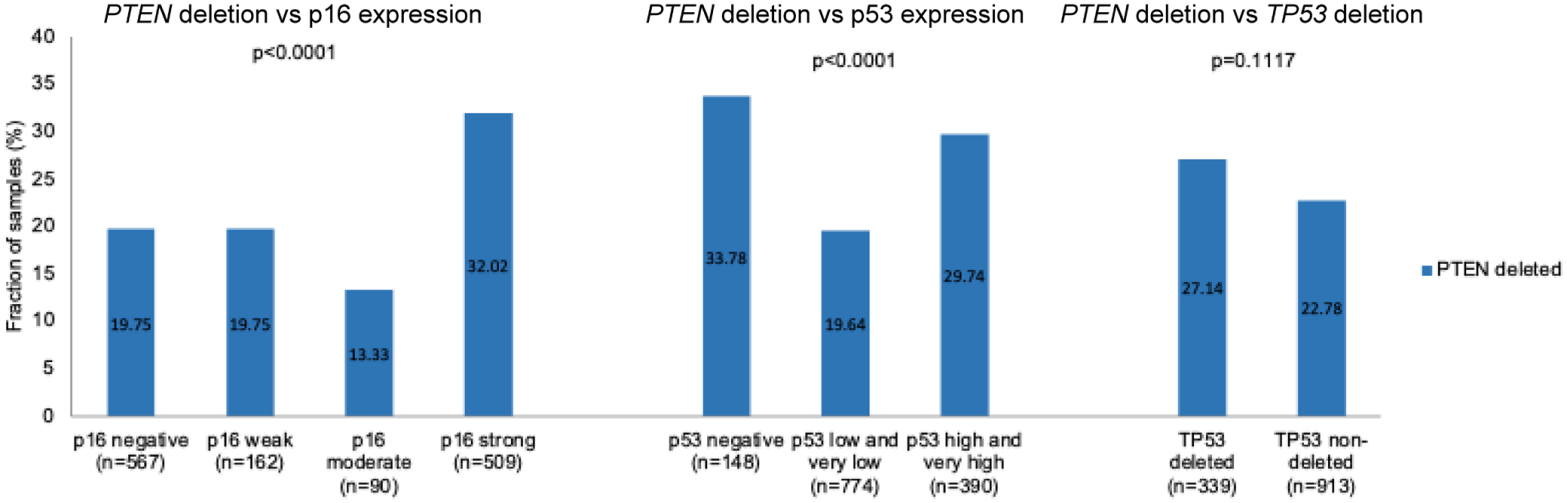
*PTEN* copy number status and p16 and p53 immunostaining and *TP53* copy number status.

Comparison of our *PTEN* FISH data with p53 and p16 data from previous studies [30, 31] revealed a statistically significant association between *PTEN* deletions and aberrant p53 and p16 immunostaining (*p* < 0.0001 each) in the subset of pT2‐4 urothelial bladder carcinomas (Figure 3). *PTEN* deletions were most common in cancers with complete lack of p53 immunostaining (33.8%; potential p53 null phenotype) followed by tumors with high and very high p53 immunostaining (29.8%). *PTEN* deletions were also more common in cancers with strong p16 immunostaining (32%) than in cancers without p16 staining (19.8%). Statistical associations were not found between *PTEN* and *TP53* deletions (*p* = 0.1117). It is of note that tumors harboring combined *PTEN* and p53 alterations are associated with poorer prognosis than tumors without alterations of these molecular markers (Figure S1).

## 4 Discussion

The successful analysis of more than 1800 urothelial bladder carcinomas showed the largest increase of *PTEN* deletion frequency within the subset of 503 non‐invasive urothelial bladder carcinomas. In these cancers, the fraction of *PTEN* deleted tumors increased from 3.1% in pTaG2 low‐grade to 4.5% in pTaG2 high‐grade and 20.7% in pTaG3 tumors. A distinct increase of *PTEN* deletions with increasing grade in non‐invasive (pTa) urothelial neoplasms has also been shown by others and us. Calderaro et al. have found an increase of *PTEN* deletions from 0% in pTaG2 low‐grade to 20% in pTG2 high‐grade tumors [23] and Cordes et al. have shown a *PTEN* deletion rate of 6% in pTaG2 low‐grade, 11% in pTaG2 high‐grade and 29% in pTaG3 urothelial bladder tumors [21]. These data are in line with data from several of our further studies describing a marked increase of diverse genomic alterations from pTaG2 low‐grade to pTaG3 cancers. For example, Gorbokon et al. have found homozygous 9p21 deletions in 9% of pTaG2 low‐grade but in 17% of pTaG3 carcinomas [34], Plage et al. have described GATA3 high‐level amplification in 0% of pTaG2 low‐grade but in 12% of pTaG3 carcinomas [35], and Kluth et al. have found *TP53* deletions in 9% of pTaG2 low‐grade but in 24% of pTaG3 carcinomas [31]. Such a sharp increase in the number of aberrations in non‐invasive tumors with its grade of malignancy is consistent with the concept that genomic changes can best accumulate over an extended period in pTa tumors as long as cancer cells do not acquire the ability to grow invasively. As soon as a urothelial carcinoma becomes invasive its life expectancy is limited because the tumor will either be cured or will lead to the patient's death.

Our finding of 23.8% deletions in 1042 successfully analyzed pT2‐4 urothelial carcinomas is in the lower range of previously published studies showing *PTEN* deletions in 22%–80% of 12–472 muscle‐invasive urothelial bladder cancers [20, 21, 23, 25, 27, 36]. Studies reporting *PTEN* deletions in ≥ 25% of urothelial carcinomas have used either LOH [27, 36], a method where chromosome 10 aneuploidy can mimic a gene loss [37, 38] or have employed FISH with a highly sensitive threshold based on findings in normal cells [20]. Using the percentage of normal cells with a loss of a *PTEN* signal as a reference might lead to overdiagnosis because the larger average size of tumor nuclei goes along with a higher likelihood of missing signals to incomplete, truncated nuclei on thin tissue sections. Next generation sequencing studies summarized in the TCGA database have identified 1%–5% homozygous deletions [39, 40, 41], which comes close to our findings of 1.2% homozygous deletions. It is an advantage of the FISH method that the gene copy numbers can be determined directly inside each cell nuclei. This makes FISH independent of possible “contaminations” by non‐neoplastic cell nuclei and polysomy or aneusomy in tumor cell nuclei. Our restrictive criteria to only call a *PTEN* deletion if fewer *PTEN* signals than centromere 10 signals occur in ≥ 60% of tumor cells are based on the assumption that a clinically relevant heterogeneity will not occur within a tumor area of 0.6 mm in diameter.

That *PTEN* deletions were not prognostic in muscle‐invasive urothelial bladder carcinomas is in line with data from two earlier studies. Tzai et al. had analyzed 71 cancers by LOH method and found no relationship to grade, stage, or overall survival [27]. In our own study, we had also not found associations with patient survival or histopathological features of increased tumor aggressiveness in a non‐overlapping set of 260 pT2‐4 urothelial carcinomas [21]. Absence of a prognostic role of aberrations of established cancer driver genes which are highly prognostic in other cancer types appears to be a characteristic feature of pT2‐4 urothelial carcinomas. It, for example, applies to p53 [30], p16 [30] and c‐MYC [42] alterations. Absence of a prognostic role of important molecular parameters is consistent with the notorious lack of morphologic prognostic features in pT2‐4 urothelial carcinomas. In contrast to other tumor entities, even histological grading and cell morphology are largely unrelated to patient prognosis in muscle‐invasive urothelial carcinomas. For example, nested type carcinomas—a muscle‐invasive bladder cancer type with a largely benign tumor cell morphology—are subject to a similarly poor prognosis as high‐grade carcinomas [43]. It has, therefore, been recommended to avoid grading in muscle‐invasive urothelial carcinomas in the WHO classifications of genitourinary carcinomas since 2004 [44].

Because *PTEN* and p53 are two important tumor suppressors whose common physiological mission is to protect the cell from degeneration, our *PTEN* data were also compared with previously collected p53 IHC and FISH data. The association of *PTEN* deletions with p53 alterations and increased p16 expression, a feature often linked to p53 inactivation, fits well with the known interactions of these genes. PTEN and p53 are components of a complex antagonistic pathway that controls mechanisms driving cell survival and cell death (summarized in [45]). For example, PTEN can stabilize p53 by complex building in the cell nucleus [46]. This improves p53 DNA binding and enforces its support for the transcription of genes involved in cell cycle control, DNA repair, apoptosis, and cellular senescence [47]. p53 can directly increase PTEN expression by binding to the *PTEN* promoter and thus support PTEN inhibition of the PIK3/AKT pathway [48]. Therefore, the combination of p53 inactivation and PTEN loss leads to a survival advantage for tumor cells and usually also to increased tumor progression [49]. That combinations of *PTEN* deletions with p53 alterations show poorer prognosis in our patient cohort is, thus, in line with the known interaction of these proteins.

The clinical significance of *PTEN* deletion in urothelial carcinomas appears to be limited to its predictive role. Several studies have shown that *PTEN* deficiency plays an important role regarding the resistance of tumors to inhibitors of the PIK3/AKT signaling pathway. For example, suppression or inactivation of PTEN is related to higher sensitivity to temsirolimus (allosteric mTORC1 inhibitor), MK‐2206 (allosteric AKT inhibitor), AZD6482 (PI3K/p110β inhibitor), ipatasertib (AKT inhibitor), and 17‐AAG (HSP90 chaperone inhibitor) that induces many degradation of proteins like HER2 and AKT [50, 51]. PTEN‐deficient cancers may also be more sensitive to PARP inhibitors [52, 53]. Studies have also shown that PTEN deficiency promotes resistance to anti‐PD1 [23] and anti‐CTLA4 [54] therapies and that inhibition of the PIK3/AKT pathway in PTEN‐deficient tumor cells could overcome the immunosuppressive tumor phenotype and the resistance to immune checkpoint inhibitors [54].

## 5 Conclusion

In this large multi‐center study, *PTEN* deletions accumulate with grade progression in non‐invasive urothelial bladder carcinomas in line with our previous study from Cordes et al. [21]. While the absence of a prognostic role of *PTEN* deletions is in line with the assumption that single markers like *PTEN* deletions, in isolated analysis, often failed to yield strong prognostic information in muscle‐invasive urothelial bladder carcinomas. However, it appears likely that the *PTEN* deletion status will be clinically important for predicting response to several treatment types.

## Author Contributions

M.K., S.M., R.S., G.S.: contributed to conception, design, data collection, data analysis, and manuscript writing. H.P., K.F., S.H., S.W., B.R., A.F., Md.M., S.S., S.E., M.L., A.H.M., H.S., M.F., M.R., M.S., K.K., T.E., S.K., N.A., J.W., H.Z., D.H.: participated in pathology data analysis, data interpretation, and collection of samples. M.K., R.S.: data analysis. M.K.: FISH analysis. M.K., R.S., T.S., G.S., S.M.: study supervision. All authors agree to be accountable for the content of the work.

## Funding

The authors have nothing to report.

## Disclosure

The authors have nothing to report.

## Ethics Statement

The use of archived remnants of diagnostic tissues for manufacturing of TMAs and their analysis for research purposes as well as patient data analysis has been approved by local laws (HmbKHG, §12) and by the local ethics committee (Ethics commission Hamburg, WF‐049/09). All work has been carried out in compliance with the Helsinki Declaration.

## Consent

The authors have nothing to report.

## Conflicts of Interest

The authors declare no conflicts of interest.

## Supporting information


**Table S1:** PTEN copy number status—separated into heterozygous and homozygous deletion—and tumor phenotype.
**Figure S1:** Combined *PTEN* and (A) p53 and (B) p16 status and patient prognosis. (A) *PTEN* and p53 normal = *PTEN* normal and p53 very low or low, *PTEN* or p53 altered = *PTEN* deleted or p53 negative or p53 high or very high, *PTEN* and p53 altered = *PTEN* deleted and p53 negative or p53 high or very high. (B) *PTEN* and p16 normal = *PTEN* normal and p16 low or moderate, *PTEN* or p16 altered = *PTEN* deletion or p16 strong or negative, *PTEN* and p16 altered = *PTEN* deletion and p16 strong or negative.

## Data Availability

All data generated or analyzed during this study are included in this published article.
