# Supplementary material for: PTEN Deletions Are Associated With Tumor Progression But Unrelated to Patient Prognosis in Muscle‐Invasive Urothelial Bladder Carcinomas: A Large Multi‐Center Validation Study on 2710 Urothelial Bladder Carcinomas
Source: Genes Chromosomes Cancer. 2026 Jan 24;65(1):e70105. doi: 10.1002/gcc.70105 (PMC12831224; doi:10.1002/gcc.70105)
Supplement: Supplementary file 1 — Table S1: PTEN copy number status—separated into heterozygous and homozygous deletion—and tumor phenotype. Figure S1: Combined PTEN and (A) p53 and (B) p16 status and patient prognosis. (A) PTEN and p53 normal = PTEN normal and p53 very low or low, PTEN or p53 altered = PTEN deleted or p53 negative or p53 high or very high, PTEN and p53 altered = PTEN deleted and p53 negative or p53 high or very high. (B) PTEN and p16 normal = PTEN normal and p16 low or moderate, PTEN or p16 altered = PTEN deletion or p16 strong or negative, PTEN and p16 altered = PTEN deletion and p16 strong or negative. [file GCC-65-e70105-s001.docx]

**Supplementary table 1. *PTEN* copy number status – separated into heterozygous and homozygous deletion - and tumor phenotype**

**Supplementary figure 1.** Combined *PTEN* and A) p53 and B) p16 status and patient prognosis. A) *PTEN* and p53 normal = *PTEN* normal and p53 very low or low, *PTEN* or p53 altered = *PTEN* deleted or p53 negative or p53 high or very high, *PTEN* and p53 altered = *PTEN* deleted and p53 negative or p53 high or very high. B) *PTEN* and p16 normal = *PTEN* normal and p16 low or moderate, *PTEN* or p16 altered = *PTEN* deletion or p16 strong or negative, *PTEN* and p16 altered = *PTEN* deletion and p16 strong or negative
